# Supplementary figures and images for: Salivary microbiomes of indigenous Tsimane mothers and infants are distinct despite frequent premastication
Source: PeerJ. 2016 Nov 3;4:e2660. doi: 10.7717/peerj.2660 (PMC5101600; doi:10.7717/peerj.2660)

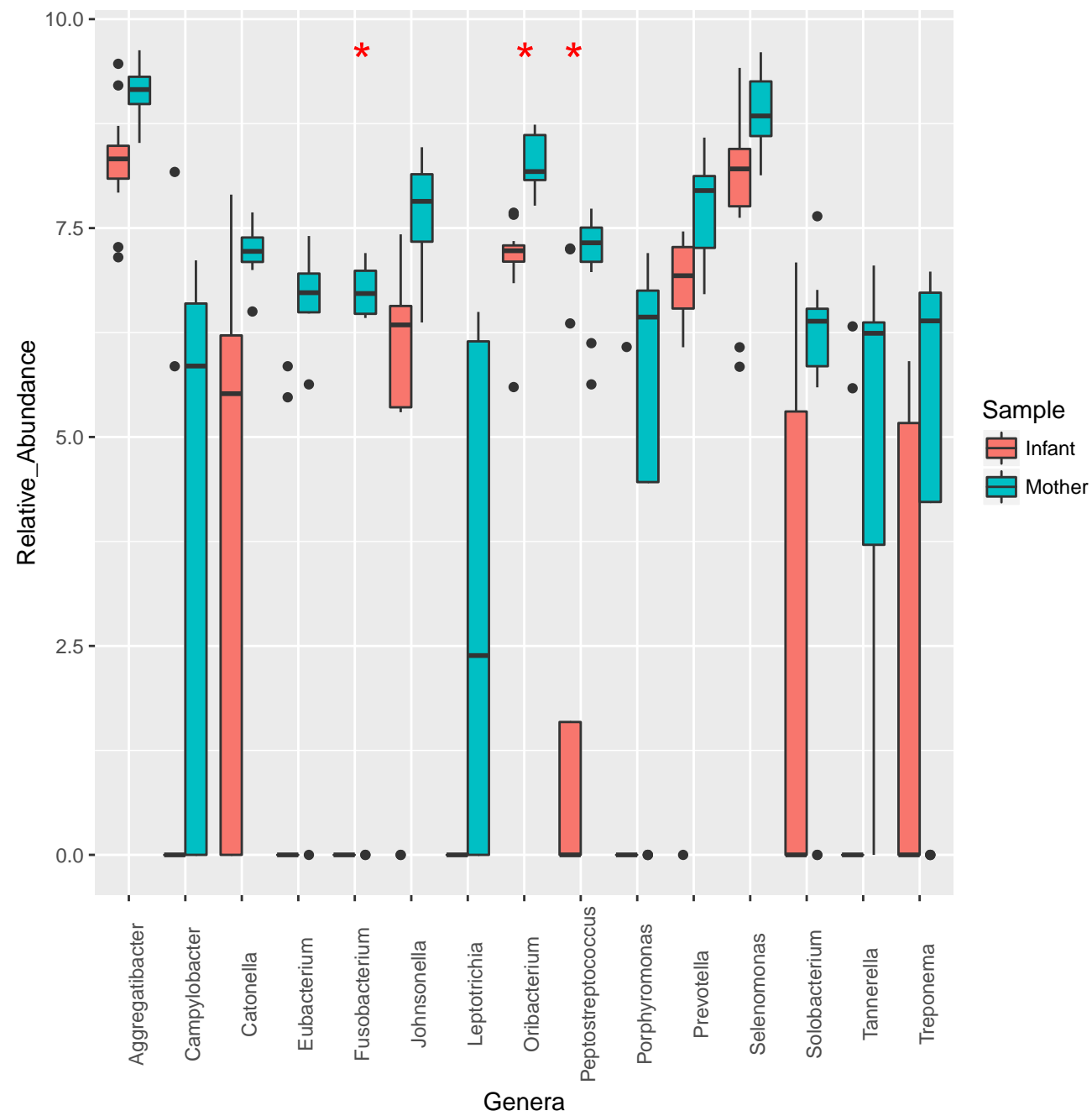

Supplement: Figure S2 — Graph depicts values in log scale (log10(x) + 10). Asterisks denote significant differences in abundance between maternal and infant samples (∗p < 0.05, ∗∗p < 0.01). [file peerj-04-2660-s002.pdf]

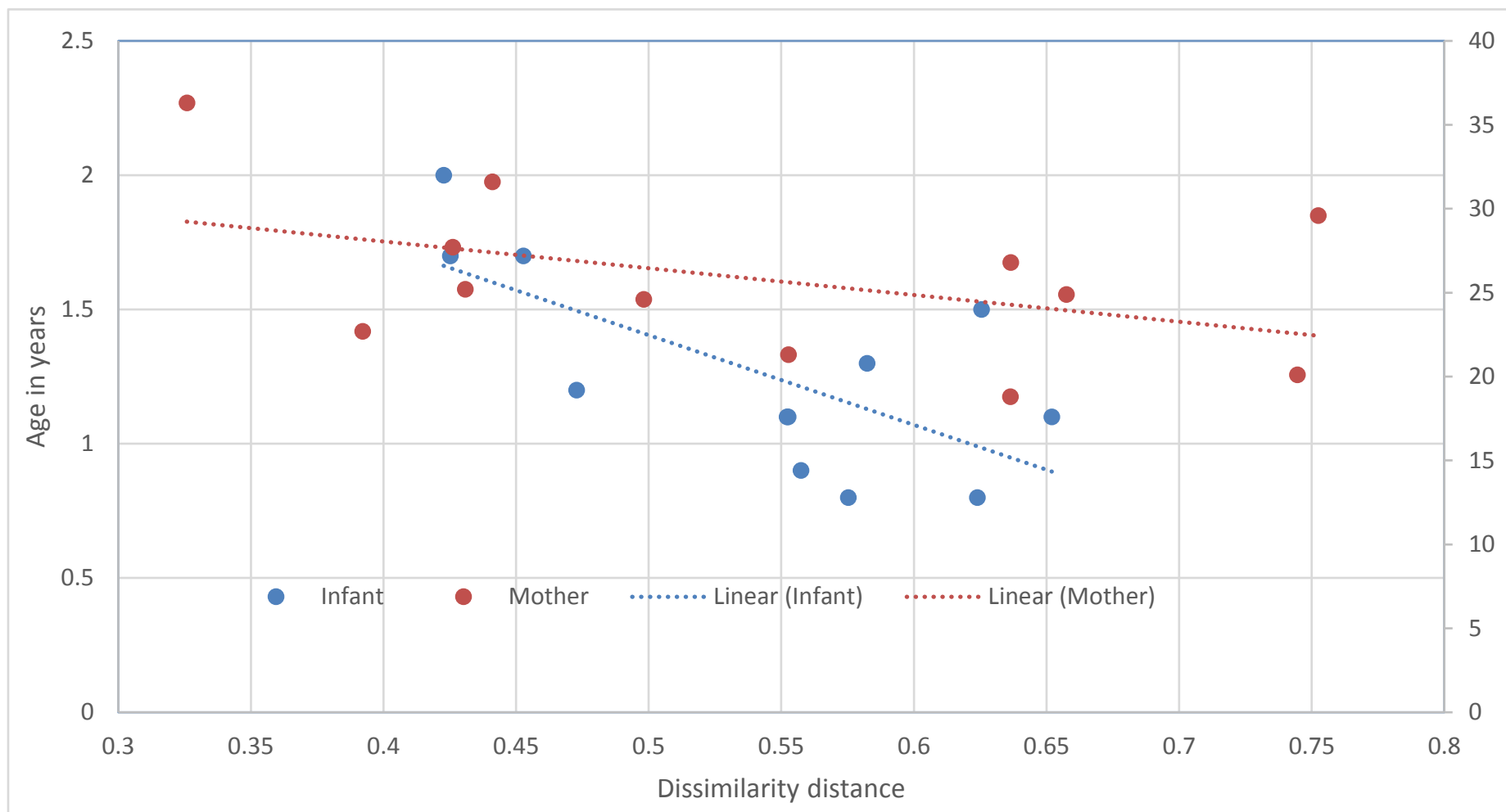

Supplement: Figure S3 — Average dissimilarity distances between each infant and all mothers are negatively correlated with infant age, r = 0.64, p = 0.025. Average dissimilarity distances between a mother and all infants are not correlated with maternal age, r = − 0.33, p = 0.293. [file peerj-04-2660-s003.pdf]
