# Supplementary material for: Salivary microbiomes of indigenous Tsimane mothers and infants are distinct despite frequent premastication
Source: PeerJ. 2016 Nov 3;4:e2660. doi: 10.7717/peerj.2660 (PMC5101600; doi:10.7717/peerj.2660)
Supplement: Figure S1 [file peerj-04-2660-s001.pdf]

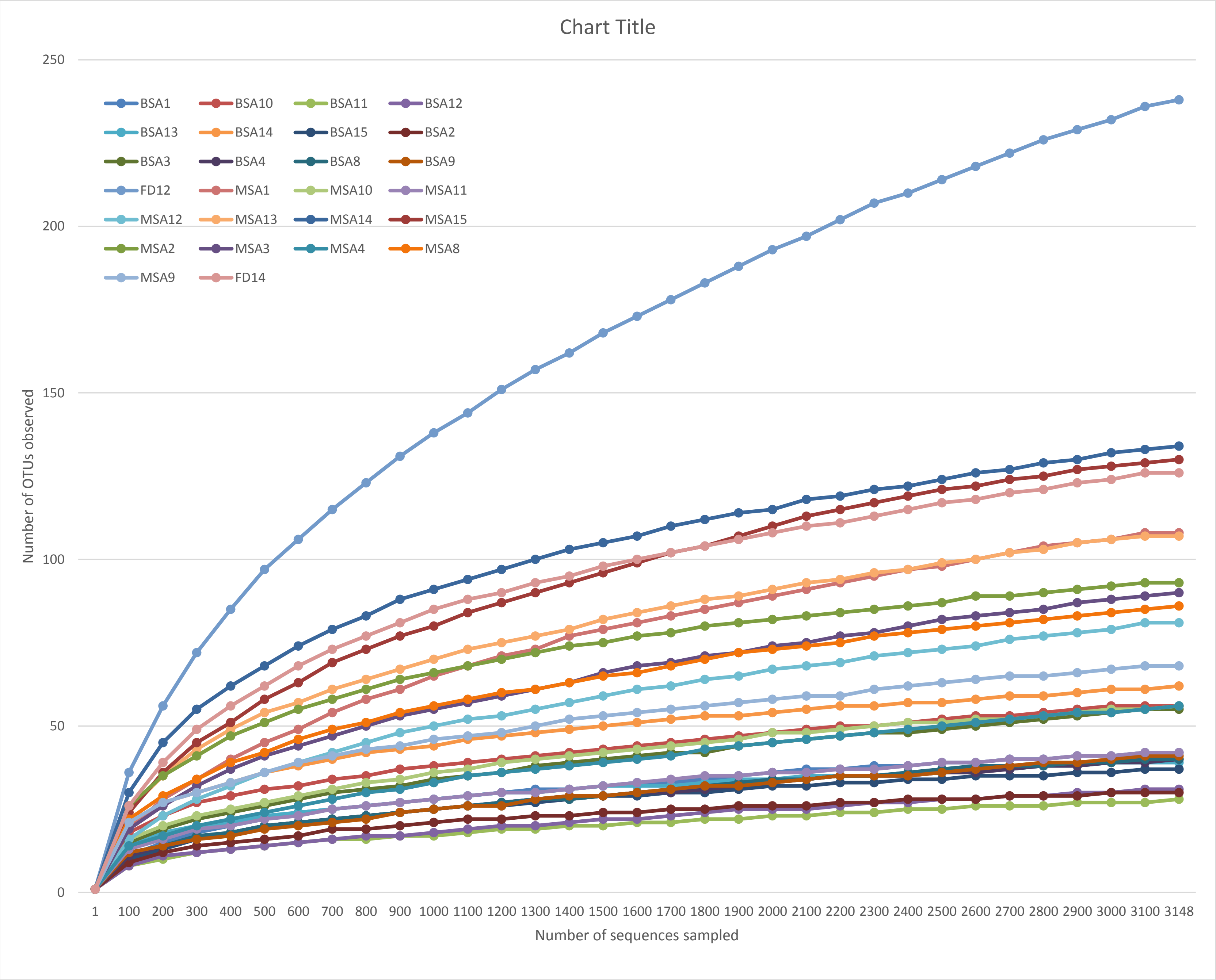
Chart Title
250
200
150
100
50
0
Number of OTUs observed
BSA1
BSA10
BSA11
BSA12
BSA13
BSA14
BSA15
BSA2
BSA3
BSA4
BSA8
BSA9
FD12
MSA1
MSA10
MSA11
MSA12
MSA13
MSA14
MSA15
MSA2
MSA3
MSA4
MSA8
MSA9
FD14
1 100 200 300 400 500 600 700 800 900 1000 1100 1200 1300 1400 1500 1600 1700 1800 1900 2000 2100 2200 2300 2400 2500 2600 2700 2800 2900 3000 3100 3148
Number of sequences sampled
